# Application of Transgalactosylation Activity of β-Galactosidase from *Kluyveromyces lactis* for the Synthesis of Ascorbic Acid Galactoside

**DOI:** 10.1007/s12010-017-2551-z

**Published:** 2017-07-13

**Authors:** Aleksandra Wojciechowska, Robert Klewicki, Michał Sójka, Katarzyna Grzelak-Błaszczyk

**Affiliations:** Institute of Food Technology and Analysis, Lodz University of Technology, Stefanowskiego 4/10, 90-924 Łódź, Poland

**Keywords:** Ascorbic acid, β-Galactosidase, *Kluyveromyces lactis*, Transgalactosylation, Lactose

## Abstract

In view of a commonly known beneficial role and low stability of ascorbic acid, many efforts are constantly undertaken to produce its improved derivatives. This paper presents results on the synthesis of ascorbic acid galactoside using transgalactosylation properties of β-galactosidase from *Kluyveromyces lactis* and lactose as a donor of galactosyl moiety. The purpose of this study was to determine the influence of selected factors (concentration and molar ratio of substrates, amount of the enzyme preparation, pH of the solution, presence of different ions) on the course of transgalactosylation reaction. Research has shown that approx. 2.5% dry matter (d.m.; 12.7 g/L) of ascorbic acid galactoside is formed under favourable conditions (50% (*w*/*v*) substrates, sodium ascorbate and lactose at the molar ratio of 1.9:1, enzyme dose of 28,600 U/100 g lactose, pH = 7.0). The addition of Mg^2+^ or K^+^ ions to the reaction medium caused an increase in the final product content (even up to approx. 3.4% d.m., 17.2 g/L), while Na^+^ or Mn^2+^ had an adverse impact on the yield. The gathered data may be valuable for cosmetic or food industry.

## Introduction

Ascorbic acid (known as vitamin C) is famous for its health benefits (e.g. stimulating collagen synthesis, preventing common cold, enhancing absorption of iron, even anti-carcinogenic activity). Among sources of this vitamin, fresh fruits and vegetables are the main ones. It is also available in many kinds of dietary supplements and frequently incorporated into food as a natural antioxidant. Unfortunately, ascorbic acid is considered a substantially labile compound [1]. Therefore, more stable ascorbyl derivatives (e.g. phosphatic [2] and fatty acid esters [3] or glucosides [4]) have been an object of interest for years.

Ascorbic acid glucosides may be synthesized by biotechnological and chemical methods. Hsieh et al. [5] used whole cells of *Aspergillus niger* (and maltose as a substrate) for this purpose. However, it seems that many researchers focused on enzymatic synthesis of derivative called 2-*O*-d-glucopyranosyl-l-ascorbic acid. Muto et al. [6] used rice seed α-glucosidase and maltose as substrate, Liu et al. [7, 8] used cyclodextrin glycosyltransferase from *Paenibacillus macerans* and maltose or maltodextrin, Zhang et al. [9] used α-cyclodextrin glucanotransferase from recombinant *Escherichia coli* and β-cyclodextrin and Kwon et al. [10] used sucrose phosphorylase from *Bifidobacterium longum* and sucrose. Other glycosidic linkages are also possible. For instance, Takahiko et al. [11] patented manufacture of 5-*O*-α-d-glucopyranosyl-l-ascorbic acid utilizing cyclodextrin glucanotransferase. 6-*O*-α-d-Glucosyl- and 6-*O*-α-d-maltosyl-ascorbic acids were identified as a result of *Bacillus stearothermophilus* maltogenic amylase activity in experiments of Bae et al. [12]. Moreover, 3-*O*-glyco-l-ascorbic acid obtained by Li and Shi [13] is an example of ascorbic acid modification on the less common, chemical way. It is worth noting that ascorbic acid glucosides can be also found in nature. They have been detected in some plants from Cucurbitaceae [14] and Solanaceae family [15] and also processed food such as kimchi (Korean traditional fermented food) [16].

Interestingly, very few literature sources concern ascorbic acid galactoside synthesis, although transgalactosylation is a similar method of modification to that one mentioned above. In that case, specific galactosidase transfers a galactosyl residue onto an ascorbic acid molecule. Hashimoto et al. [17] used *Candida guilliermondii* H-404 α-galactosidase and melibiose or phenyl-alpha-galactoside (as a donor substrate) for production of 6-*O*-α-d-galactopyranosyl-l-ascorbic acid. Kitahata et al. [18] proved utility of raffinose as an alternative substrate in this process. Synthesis of 6-*O*-β-d-galactopyranosyl-l-ascorbic acid was described by Hong et al. [19] as the effect of transgalactosylation properties of β-galactosidase from *Aspergillus oryzae*. Presumably, the exact same reaction was patented earlier by Donpou et al. [20] and another one by Kawanaka et al. [21] but with β-galactosidase from *Bacillus circulans*. On the other hand, Shimono et al. [22] obtained 2-*O*-β-d-galactopyranosyl-l-ascorbic acid utilizing also β-galactosidase from *A. oryzae*, but 5,6-isopropylidene-l-ascorbic acid as an acceptor. All processes based on β-galactosidase activity required lactose as a donor substrate. Therefore, it should also be mentioned that there is an enormous potential for using whey (a by-product from the dairy industry) as a cheap source of substrate (lactose). It has already been done in the case of other transgalactosylation product synthesis (e.g. galactooligosaccharides) [23].

Research has shown that ascorbic acid glucosides and galactosides possess a number of beneficial properties similar to that of ascorbic acid. Mainly due to its antioxidant capacity, these derivatives can suppress browning of green tea beverage [24], reduce lipid oxidation in food products [25] or extend the shelf life of harvested fruits and vegetables [26]. Free radical scavenging and metal chelating activity [27] as well as skin-lightening effect or enhancement of collagen synthesis [28, 29] are extremely valuable for cosmetic industry. 6-*O*-β-d-Galactopyranosyl-l-ascorbic acid may also be applied as an active ingredient of dentrifices [30] or ophthalmic solutions [31]. There is no available data comparing properties of glucose and galactose derivatives of ascorbic acid, but it is possible that the presence of galactose instead of glucose in the molecule may change its biological activity.

The purpose of the study was to identify the impact of selected factors on the course of the synthesis reaction of ascorbic acid galactoside-utilizing transgalactosylation activity of β-galactosidase from *Kluyveromyces lactis*. The content and molar ratio of substrates (lactose and sodium ascorbate) in the initial solution, the enzyme dose pH and presence of selected salts in different concentrations were the subject of analysis. It should be stressed that no data reports on the use of galactosidase from this species as a source for ascorbic acid derivatization. Furthermore, no available literature concerning synthesis of ascorbic acid β-galactoside is focused on the effect of different parameters on this process.

## Materials and Methods

### Materials

Lactozym Pure 2600L, β-galactosidase, EC 3.2.1.23 from *K. lactis* was purchased from Novozymes A/S (Bagsvaerd, Denmark). Lactose and ascorbic acid sodium salt were purchased from Stanlab (Lublin, Poland). A HPLC standard, 2-*O*-α-d-glucopyranosyl-l-ascorbic acid, was purchased from ThermoFisher GmbH (Karlsruhe, Germany). NaOH was purchased from Eurochem BGD Sp. z.o.o. (Tarnów, Poland). NaCl and KCl were purchased from POCH (Gliwice, Poland). MgCl_2_·6H_2_O, MnCl_2_·4H_2_O and H_2_SO_4_ were purchased from Chempur (Piekary Śląskie, Poland). A reagent for determination of glucose concentration was purchased from BioMaxima S.A. (Lublin, Poland).

### Synthesis of Ascorbic Acid Galactoside

#### Synthesis of Ascorbic Acid Galactoside in Solutions with Different Contents of Dry Mass

β-Galactosidase in the amount of 28,600 U/100 g of lactose was added to 100 mL of solution containing 20, 30, 40 or 50 g of the mixture of sodium ascorbate and lactose at a molar ratio of 1.9:1. The reaction was carried out at 37 ± 1 °C, pH 6.9–7.0 (adjusted with 0.25 M NaOH solution), for 9 h. Samples of the reaction mixture (1 mL) were collected every 60 min.

#### Synthesis of Ascorbic Acid Galactoside in Solutions with Various Molar Ratios of Substrates

β-Galactosidase in the amount of 28,600 U/100 g of lactose was added to 100 mL of solution containing 50 g of the mixture of sodium ascorbate and lactose at the following molar ratio of 1:0.184, 1:0.303, 1:0.526, 1:0.731, 1:1 and 1:1.9. The reaction was carried out at 37 ± 1 °C, pH 6.9–7.0 (adjusted with 0.25 M NaOH solution), for 9 h. Samples of the reaction mixture (1 mL) were collected every 60 min.

#### Synthesis of Ascorbic Acid Galactoside Acid Using Different Enzyme Doses

The following amounts of β-galactosidase were added to 100 mL of a solution containing 50 g of the mixture of sodium ascorbate and lactose (at the molar ratio of 1.9:1): 7130, 14,200, 21,400, 28,600, 35,600, 42,800 and 49,900 U/100 g of lactose. The reaction was carried out at 37 ± 1 °C, pH 6.9–7.0 (adjusted with 0.25 M NaOH solution), for 9 h. Samples of the reaction mixture (1 mL) were collected every 60 min.

#### Synthesis of Ascorbic Acid Galactoside in Solutions with Different pH Values

β-Galactosidase in the amount of 28,600 U/100 g of lactose was added to 100 mL of solution containing 50 g of the mixture of sodium ascorbate and lactose at the molar ratio of 1.9:1. The reaction was carried out at 37 ± 1 °C for 9 h. The pH value of the solution was adjusted respectively to 6.4–6.5, 6.9–7.0, 7.4–7.5, 7.9–8.0 and 8.4–8.5 (with 0.25 M NaOH). Samples of the reaction mixture (1 mL) were collected every 60 min.

#### Synthesis of Ascorbic Acid Galactoside in the Presence of Selected Salts of Different Concentrations

β-Galactosidase in the amount of 28,600 U/100 g of lactose was added to 100 mL of solution containing 50 g of the mixture of sodium ascorbate and lactose at the molar ratio of 1.9:1. The reaction was carried out in presence of selected salt (0.1 and 1 M NaCl; 0.1, 0.5 and 1 M KCl; 0.1, 0.5, 0.75 and 1 M MgCl_2_; 0.1 M MnCl_2_) at 37 ± 1 °C, pH 6.9–7.0 (adjusted with 0.25 M NaOH solution), for 9 h. Samples of the reaction mixture (1 mL) were collected every 60 min.

### HPLC Determination of Concentration of Ascorbic Acid and Its Galactoside

A sample (1 mL) of the reaction mixture was collected for the analysis and introduced to approx. 30 mL of boiling distilled water. Boiling was maintained for 1 min to inactivate the enzyme. The solution was cooled down to room temperature and placed in a 100-mL measuring flask. Then, it was filled up with water, mixed and filtered. Six millilitres of filtrate was passed through a column containing cationite for desalination. The first fraction (3 mL) was removed, and another 3 mL was taken for further investigation. HPLC analysis was performed on Shimadzu Prominence system fitted with a LC-20AD pump, a SIL-20AC autosampler, a CTO-10AS oven and a SPD-20AV UV detector (Shimadzu, Kyoto, Japan). Separation conditions were as follows: column, Aminex HPX-87H (Bio-Rad, Richmond, CA, USA); mobile phase, 0.005 H_2_SO_4_; flow rate, 0.6 mL/min; and temperature, 40 °C. Detection was carried out at 210 nm wavelength. Due to high structural similarity, commercially available ascorbic acid glucoside (2-*O*-α-d-glucopyranosyl-l-ascorbic) was used as a standard for the determination of the concentration of galactosyl derivative of ascorbic acid.

### HPLC Determination of Saccharide Content

A 1-mL sample of the reaction mixture was collected and introduced to approx. 30 mL of boiling distilled water. Boiling was maintained for 1 min. After cooling down to a room temperature, the solution was transferred to a 100-mL measuring flask. Then, it was filled up with water, mixed and filtered. The filtrate was passed through a column containing cationite and anionite. The first fraction (3 mL) was discarded and another 3 mL was taken for HPLC analysis. The concentration of galactooligosaccharides (GOS), lactose, glucose and galactose were determined with the use of Aminex HPX87C column from Bio-Rad (Hercules, CA, USA): mobile phase, water; flowrate, 0.5 mL/min; temperature, 85 °C; and detection, KNAUER RI Detector 2300 (Berlin, Germany). Lactose standard was used for the estimation of GOS content.

### MS Analysis for Post-reaction Mixture

A diluted sample of post-reaction mixture was directly introduced to the detector at a flow rate of 10 μL/min. QExactive Orbitrap (ThermoScientific, Waltham, MA, USA) was used for the mass spectrum analysis in negative-ion mode. Parameters of the source are the following: ion spray voltage, 3.00 kV; capillary temperature, 300 °C; and sheath gas and auxiliary gas flow rate, 30 and 5 units/min.

### Purification of the Product Using Preparative Chromatography and Ion Exchange

A portion of reaction mixture was passed through a cationite-filled column (elution with water) and then concentrated on a rotary evaporator to approx. 30–35 °Bx. A sample in the amount of 10 mL was introduced into a preparative chromatography column (length, 1 m; diameter, 33 mm) containing ion-exchange resin (Dowex 50WX4 hydrogen form, 100–200 mesh, Alfa Aesar GmbH, Karlsruhe, Germany). Separation was carried out using water as a mobile phase, at a flow rate of 4.5 mL/min and temperature 40 °C. KNAUER Variable Wavelength Monitor (*λ* = 210 nm) and KNAUER Differential-Refractometer (Berlin, Germany) were used for the detection. Fractions (9 mL each) with the highest content and purity of the product were collected during a preparative run, then combined and concentrated to 5 mL. Subsequently, sample was passed through an anionite-filled column to remove nonacidic impurities (collected fraction was discarded). The exchanger filling was rinsed with 0.1% NaOH solution. The received fraction was introduced into a cationite-filled column. Collected fraction was concentrated on a rotary evaporator. The above procedure was repeated several times until a sufficient amount of substance was obtained. After all, sample was frozen and subjected to the lyophilization process (Alpha 1–2 LD Plus, Martin Christ Gefriertrocknungsanlagen GmbH, Osterode am Harz, Germany).

### NMR Analysis of Purified Product

^1^H NMR and ^13^C NMR analyses were performed on Bruker Avance II 700 MHz UltraShield Plus spectrometer (Bruker BioSpin, Billerica, MA, USA). Sample of purified and dry product was dissolved in D_2_O. Acetone was added as the internal standard. The analysis parameters were as follows: temperature, 300 K; sweep frequency, 14.2 MHz; 0.05 Hz line broadening; and number of scans, 64. TopSpin software was used for data acquisition and processing.

### Determination of β-Galactosidase Activity

The unit of activity (U) is defined as the amount of enzyme that releases 1 μmol of glucose in 1 min, in the following conditions: 4.75% (*w*/*v*) of lactose (sodium–potassium phosphate buffer, pH 6.88), temperature of 37 °C and reaction time at 30 min [32].

In phosphate buffer (pH 6.88), 4.75% (*w*/*v*) lactose and 1 mL/L Lactozym Pure 2600L solutions were prepared; 9.6 mL of lactose solution and 0.4 mL of solution of the enzyme preparation were mixed and incubated for 30 min at 37 °C. A sample of the reaction mixture (4 mL) was taken and introduced into 5 mL of boiling water. Boiling was maintained for 3 min. After cooling down to a room temperature, the solution was transferred to a 10-mL measuring flask. Then, it was filled up with water, mixed and filtered. Ten microlitres of the solution was added to 1000 μL of the reagent from BioMaxima S.A. for the determination of glucose concentration. The standard sample contained 10 μL of glucose standard, and reagent sample contained only 1000 μL of the reagent. All samples were incubated for 10 min at 25 °C. After this time, the absorbance was read (*λ* = 500 nm) for test samples and standard sample, against the reagent sample.

## Results and Discussion

### Identification of the Reaction Product

**Fig. 1:**
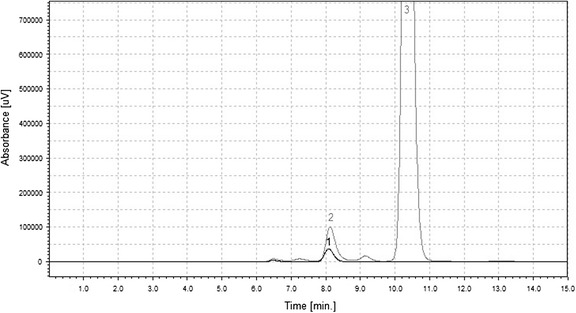
A typical chromatogram (HPLC-UV) for the post-reaction mixture and the standard (2-*O*-alpha-d-glucopyranosyl-l-ascorbic acid). Peaks *1*, standard (8.09 min); *2*, galactosyl derivative of ascorbic acid (8.13 min); and *3*, ascorbic acid (10.35 min)

**Fig. 2:**
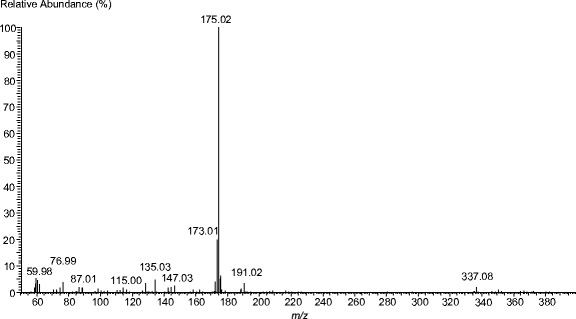
A mass spectrum for the post-reaction mixture; signal 337.1 *m/z* (negative mode)—galactosyl derivative of ascorbic acid (molar weight, 338), signal 175.0 *m/z* (negative mode)—ascorbic acid (molar weight, 176.1)

The product of β-galactosidase transgalactosylation activity strictly depends on donor and acceptor introduced into the reaction medium. The most popular donor of galactosyl residue is lactose. When lactose (or its derivative) acts as an acceptor, different galactooligosaccharides (GOS) might be obtained [23]. Other molecules possessing a hydroxyl group can be used as acceptor (e.g. sugars such as fructose [33], polyols such as glycerol [34], mannitol [35] and polyhydroxy acids such as gluconic acid [36]). In this case, the galactosyl moiety from lactose was transferred onto an ascorbic acid molecule as a result of activity of β-galactosidase produced by *K. lactis*. HPLC-UV analysis (Fig. 1) of the post-reaction mixture proved the presence of compound with a retention time similar to commercially available ascorbic acid derivative (2-*O*-α-d-glucopyranosyl-l-ascorbic). In addition, MS spectrum (Fig. 2) contains a signal at 337.1 *m/z* (negative mode) which corresponds to the molar mass of ascorbic acid galactoside (338 g/mol).

Depending on the source (microorganisms), β-galactosidase may be highly selective in transgalactosylation reaction. GOS synthesized using enzymes from *A. oryzae* or *Sulfolobus solfataricus* reveal mainly β(1 → 6) and β(1 → 4) linkages [37], while applying β-galactosidase from *K. lactis* allows to obtain GOS with prevalent β(1 → 6) linkage [38]. Moreover, other mentioned acceptors were galactosylated also at their primary hydroxyl groups utilizing *K. lactis* enzyme [33, 34]. Ascorbic acid galactosylation has already been carried out with β-galactosidase from *A. oryzae* [19] and *B. circulans* [20, 21], and the product was identified as 6-*O*-β-d-galactopyranosyl-l-ascorbic acid. Synthesis of 2-*O*-β-d-galactopyranosyl-l-ascorbic acid was possible only after locking preferential hydroxyl groups—5,6-isopropylidene-l-ascorbic acid was used as an acceptor in transgalactosylation reaction [22]. The product of presented transgalactosylation reaction was separated from the post-reaction mixture and subjected to NMR analysis (Table 1). The ^13^C NMR signals for examined substance were similar to data obtained for ascorbic acid derivative with β(1 → 6) bond [20] and different from these characteristic for derivative with β(1 → 2) bond [22]. Considering these facts, the main derivative in presented research was probably 6-*O*-β-d-galactopyranosyl-l-ascorbic acid.

**Table 1:** Signals for ^1^H NMR and ^13^C NMR spectra of ascorbic acid galactoside

^1^H NMR (*δ*, ppm)	^13^C NMR (*δ*, ppm)
3.55 (dd, *J* = 7.9, 9.9 Hz, 1H)	72.34
3.65 (dd, *J* = 3.4, 9.9 Hz, 1H)	70.43
3.70 (dd, *J* = 4.4, 8.0 Hz, 1H)	60.68
3.75 (dd, *J* = 4.3, 11.7 Hz, 1H)	69.78
3.79 (dd, *J* = 7.9, 11.7 Hz, 1H)	75.76
3.89 (dd, *J* = 6.0, 10.8 Hz, 1H)	75.79
3.92 (d, *J* = 3.5 Hz, 1H)	68.86
4.06 (dd, *J* = 7.1, 10.8 Hz, 1H)	74.86
4.27 (td, *J* = 1.8, 6.1 Hz, 1H)	117.56
4.45 (d, *J* = 7.9 Hz, 1H)	102.90
5.04 (d, *J* = 1.9 Hz, 1H)	154.96
	172.86

### The Influence of Selected Factors on the Course of the Reaction

**Fig. 3:**
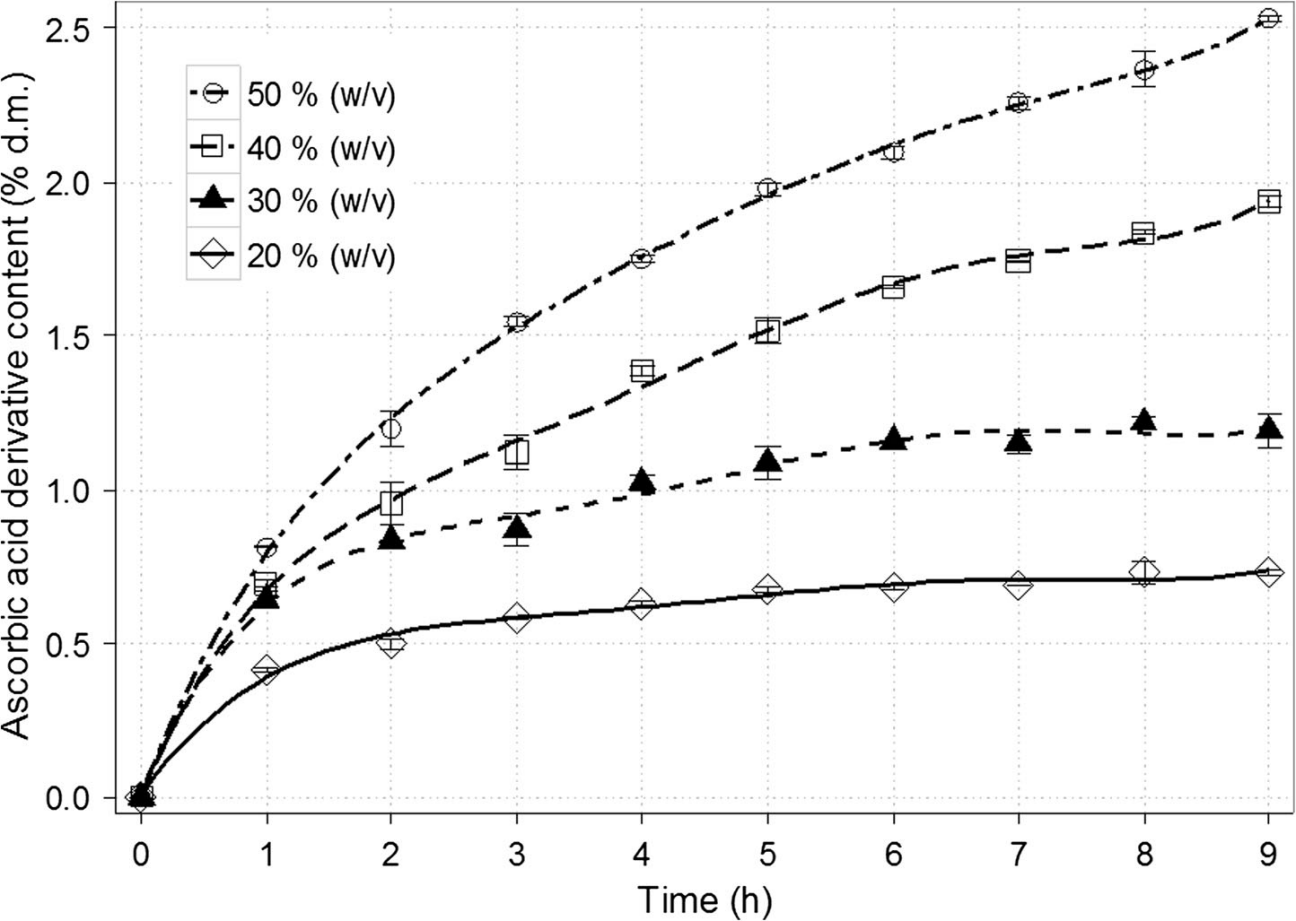
Time course for the synthesis of galactosyl derivative of ascorbic acid depending on the initial dry matter content (% *w*/*v*). The remaining reaction conditions: sodium ascorbate and lactose at the molar ratio of 1.9:1; the enzyme dose (β-galactosidase from *Kluyveromyces lactis*) of 28,600 U/100 g lactose; temp., 37 ± 1 °C; and pH 6.9–7.0

The first factor analysed was the initial content of dry mass (Fig. 3). Increasing the concentration of substrates in baseline solution from 20% (*w*/*v*) to 50% (*w*/*v*) caused corresponding increase in ascorbic acid derivative content from 0.73 to approx. 2.5% of dry matter (d.m.). Obviously, due to higher initial dry mass content, the final amount of product changed more significantly (1.46 and 12.6 g/L, respectively). This correlation was commonly observed in studies concerning galactooligosaccharide synthesis using transgalactosylation activity of β-galactosidase. GOS formation reduces with decreasing lactose concentration. For instance, Neri et al. [39] obtained 26.1% GOS from an initial solution with 50% (*w*/*v*) lactose and merely 11.2% GOS from a solution diluted to 5% (*w*/*v*). As a matter of fact, at low initial concentrations (or advanced conversion) of lactose, water becomes a more competitive acceptor. This means preferential hydrolysis of lactose rather than transgalactosylation reactions [39, 40]. However, when sorbitol was introduced as galactosyl acceptor and the dry mass content was increased beyond 35% d.m., the opposite occurred. The application of higher concentration resulted in intensified inhibitory effect of transgalactosylation products [41]. It is worth mentioning that experiments with initial solution containing above 50% (*w*/*v*) of combined lactose and sodium ascorbate were limited because of substrate solubility.

**Fig. 4:**
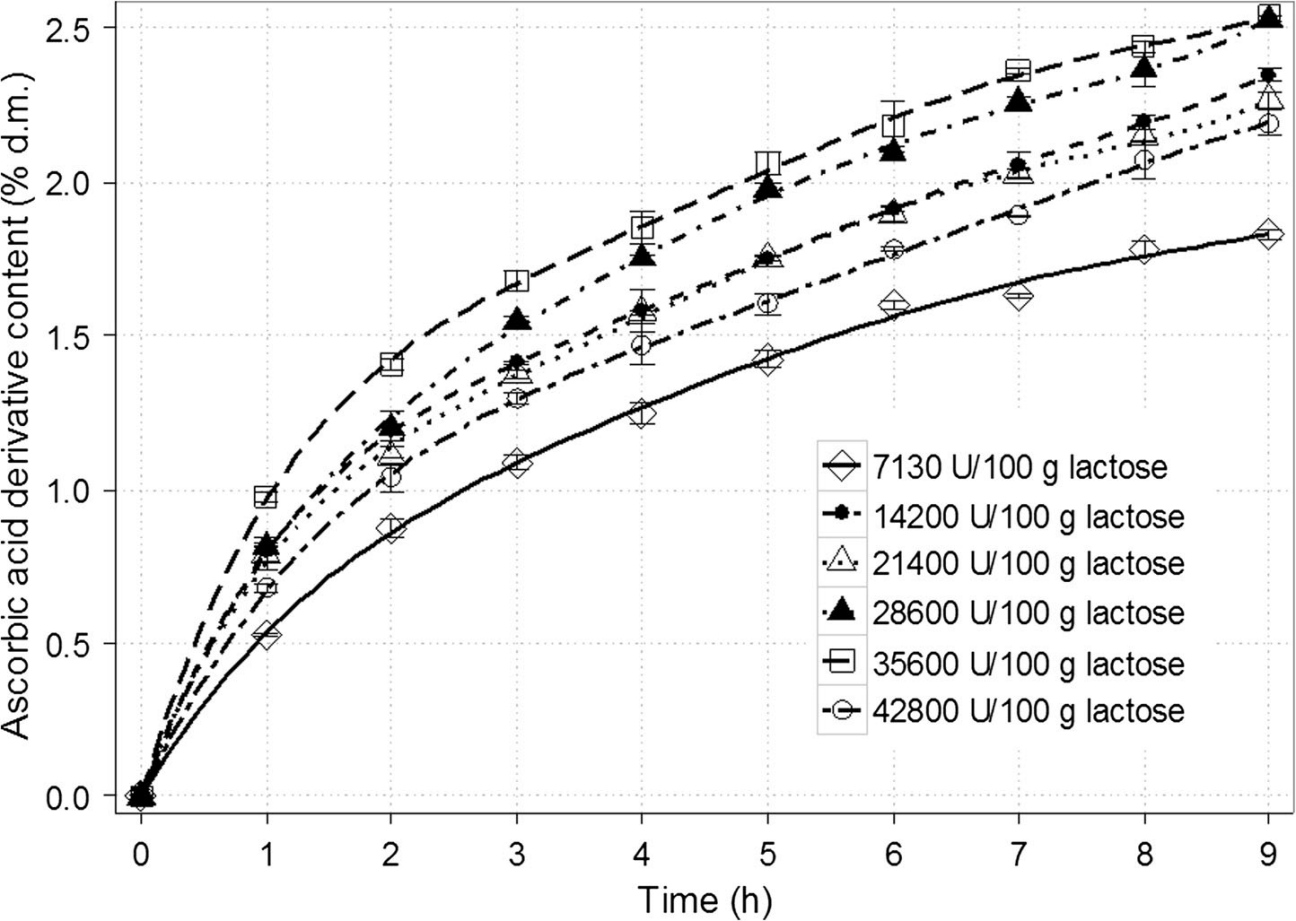
Time course for the synthesis of galactosyl derivative of ascorbic acid in a 50% (*w*/*v*) solution depending on the dose of the enzyme (β-galactosidase from *Kluyveromyces lactis*). The remaining reaction conditions: sodium ascorbate and lactose at the molar ratio of 1.9:1; temp., 37 ± 1 °C; and pH 6.9–7.0

Another important aspect for selecting optimum conditions of this type of reaction is the right dose of the enzyme preparation (Fig. 4). In this case, Lactozym Pure 2600L containing β-galactosidase from *K. lactis* was used. The maximum final contents of ascorbic acid galactoside were observed for doses of 28,600 and 35,600 U/100 g lactose (2.53 and 2.54% d.m., respectively). Indeed, the reaction for the amount of 35,600 U/100 g lactose was slightly accelerated at the beginning. However, in the case of ascorbic acid galactoside, even a significant change in the enzyme dose did not contributed to visible differences in the course of reaction. Generally, the increase of enzyme dose leads to shortening the time of transgalactosylation reaction and simultaneously further-reaching hydrolysis of transgalactosylation products [35, 42]. Considering formation of GOS, Martínez-Villaluenga et al. [42] achieved the highest trisaccharide content with the lowest tested amount of Lactozym 3000L HP G (β-galactosidase from *K. lactis*). The maximum disaccharide yield was noted in the experiment with the highest dose, but in shorter time. However, the value diminished after that time (due to the hydrolysis mentioned above). Studies on synthesis of galactosyl mannitol derivative (with the same enzyme preparation) performed by Klewicki et al. [35] demonstrated that raising the enzyme dose from an optimum of 11,400 LAU/100 g lactose to 22,800 caused almost a 7% drop in the highest concentration of product. In the presented research, this effect is not significant. The maximum amount of ascorbic acid derivative is presumably low enough to maintain hydrolysis of lactose as a privilege process. For economic reasons, the dose of 28,600 U/100 g lactose was chosen for further research.

**Fig. 5:**
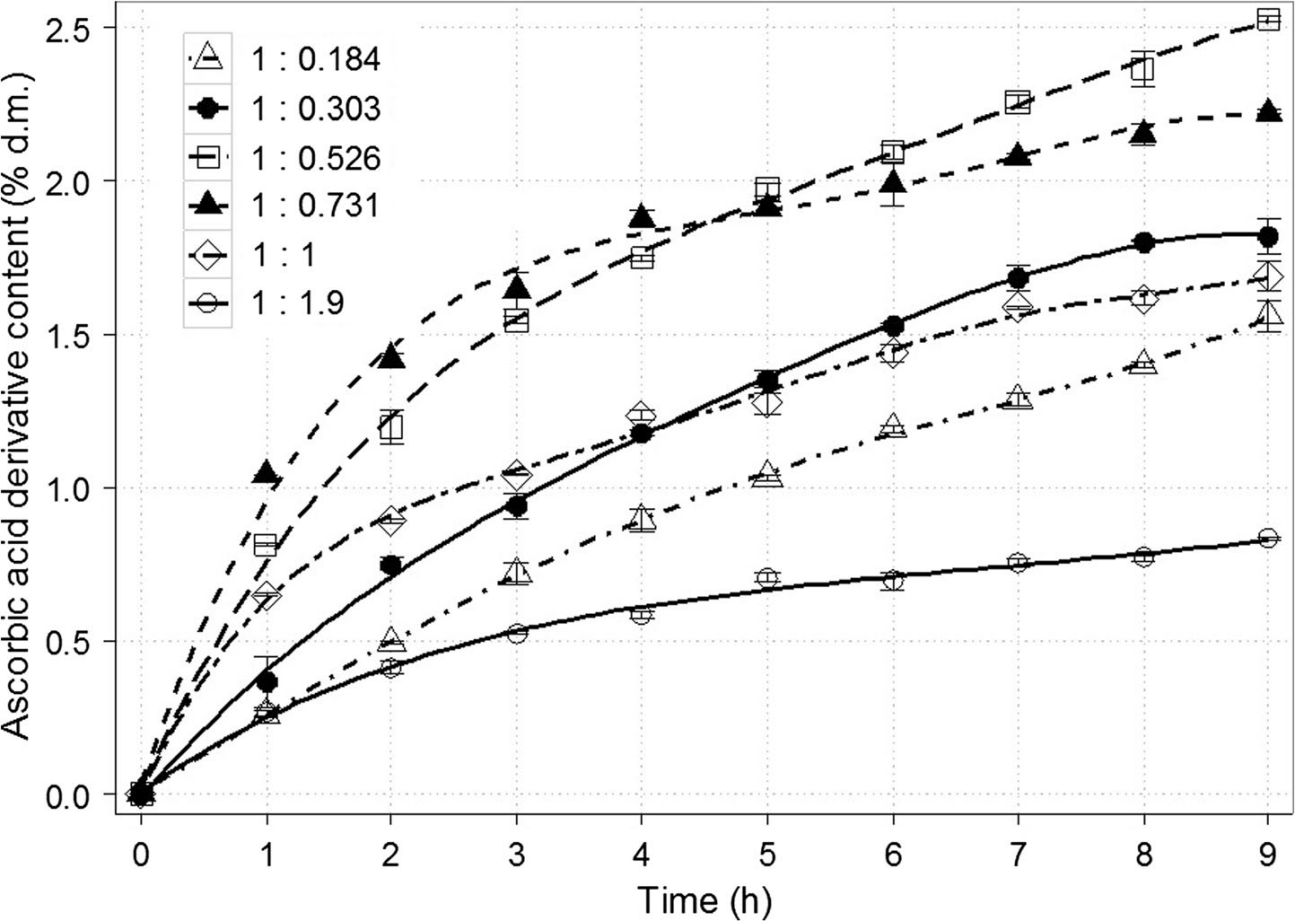
Time course for the synthesis of galactosyl derivative of ascorbic acid in a 50% (*w*/*v*) solution depending on the applied molar ratio of substrates (sodium ascorbate/lactose). The remaining reaction conditions: the enzyme dose (β-galactosidase from *Kluyveromyces lactis*) of 28,600 U/100 g lactose; temp., 37 ± 1 °C; and pH 6.9–7.0

Subsequent analysis referred to the molar ratio of acceptor and donor of galactosyl moiety (Fig. 5). The initial tested variant (sodium ascorbate and lactose introduced at a molar ratio of 1:0.526) was selected on the basis of previous research concerning galactosyl derivative of gluconic acid [36]. Comparing the concentration of ascorbic acid galactoside (as % of dry mass) in the post-reaction mixture, that ratio proved to be the best. Nevertheless, the reaction for 1:0.731 M ratio seemed to run more rapidly on the initial stage. Increasing or decreasing the share of one of the substrates caused noticeable reduction in the derivative formation. A threefold drop in final content (0.83% d.m.) was noted for the ratio of 1:1.9. On the other hand, synthesis of galactosyl-glycerol carried out by Wei et al. [34] turned out to be most successful when glycerol and galactose were used at a molar ratio from 5:1 to 10:1. The larger amount of glycerol probably inhibited enzyme activity due to competing with the water.

**Fig. 6:**
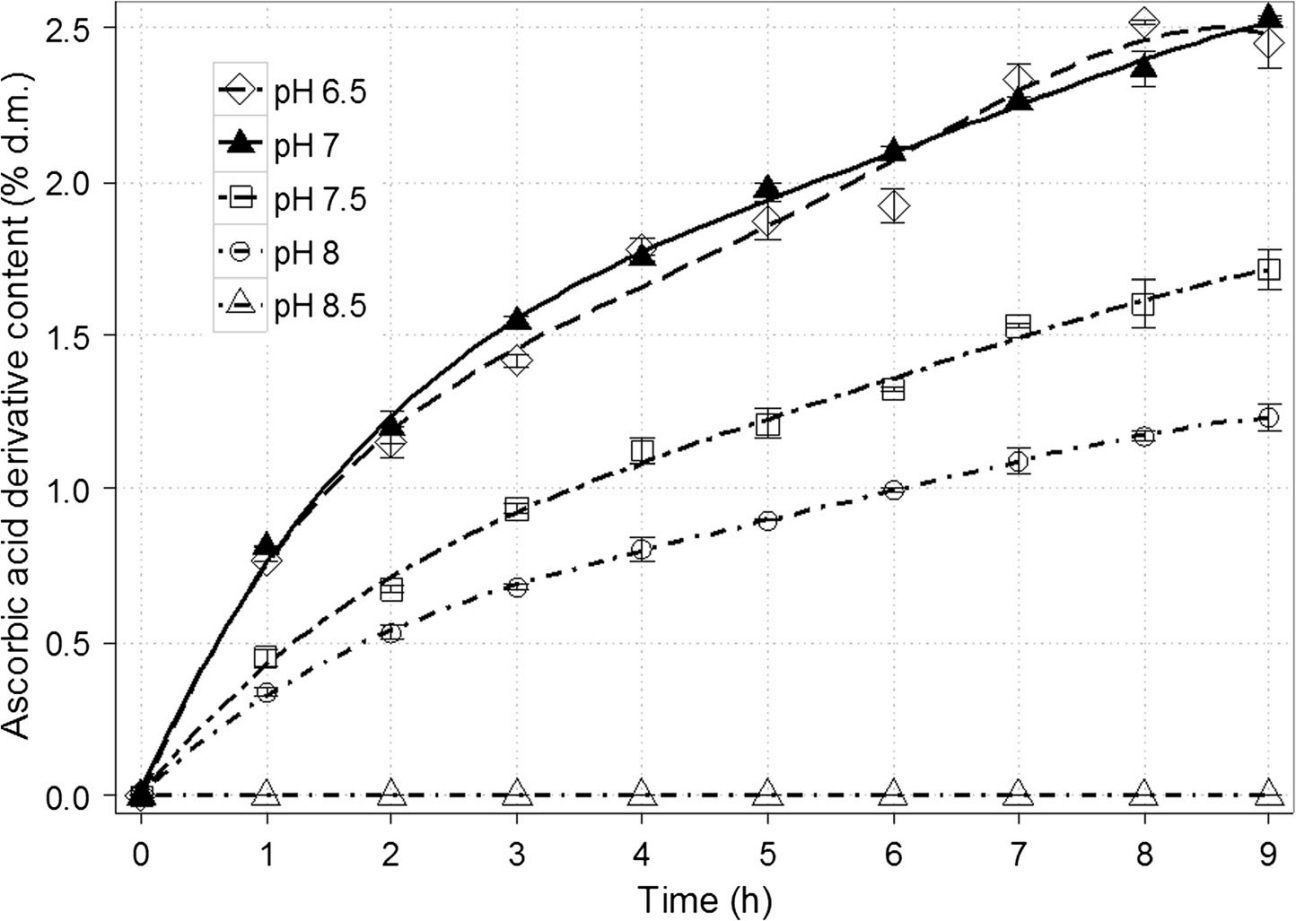
Time course for the synthesis of galactosyl derivative of ascorbic acid in a 50% (*w*/*v*) solution depending on the pH of the solution. The remaining reaction conditions: sodium ascorbate and lactose at the molar ratio of 1.9:1; the enzyme dose (β-galactosidase from *Kluyveromyces lactis*) of 28,600 U/100 g lactose; and temp., 37 ± 1 °C

The effect of the solution pH on β-galactosidase activity was also under investigation. First of all, this factor has an impact on the structure or conformation of enzyme. Optimal pH value for β-galactosidase from *K. lactis* is from 6.5 to 7 [43]. However, this range concerns rather hydrolytic properties of β-galactosidase and may be distinct for the transgalactosylation. In the case of ascorbic acid galactoside formation, the reaction proceeded most efficiently for pH of 7 and 6.5 (Fig. 6). The derivative concentration diminished approx. twofold (to 1.23% d.m.) when pH of the solution was maintained at the level of 8, whereas no product was identified for pH of 8.5. In contrast to these findings, a slightly higher content of gal-sorbitol was observed due to a rise of pH to 9 (compared with optimal pH range between 6.5 and 7.0) [41]. Increasing pH value resulted presumably in prevailing transgalactosylation activity resulting from modifications within the enzyme active site [44]. Moreover, pH influences the stability of ascorbic acid and its derivatives. For instance, the optimal pH to preserve most of ascorbic acid 2-glucoside in a cosmetic preparation is equal to 6.4 [27].

**Fig. 7:**
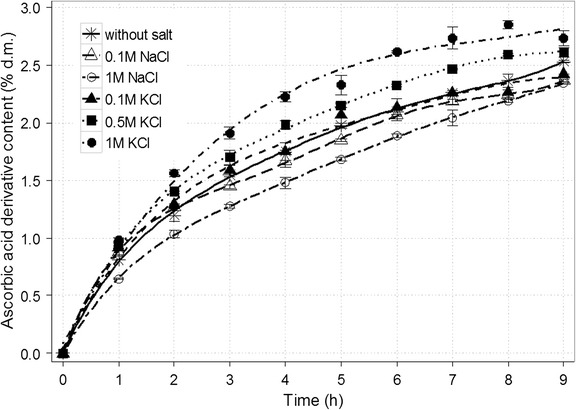
Time course for the synthesis of galactosyl derivative of ascorbic acid in a 50% (*w*/*v*) solution depending on the presence of selected salts in various concentrations. The remaining reaction conditions: sodium ascorbate and lactose at the molar ratio of 1.9:1; the enzyme dose (β-galactosidase from *Kluyveromyces lactis*) of 28,600 U/100 g lactose; temp., 37 ± 1 °C; and pH 6.9–7.0

**Fig. 8:**
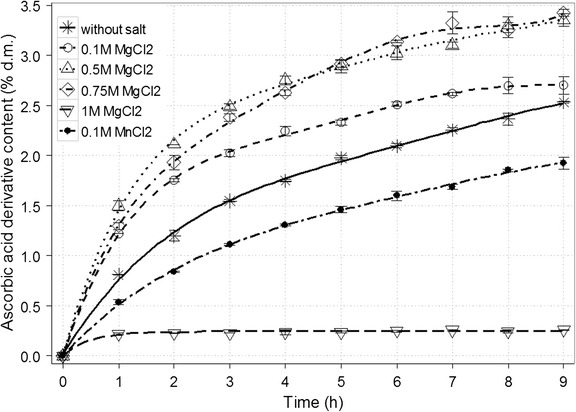
Time course for the synthesis of galactosyl derivative of ascorbic acid in a 50% (*w*/*v*) solution depending on the presence of selected salts in various concentrations. The remaining reaction conditions: sodium ascorbate and lactose at the molar ratio of 1.9:1; the enzyme dose (β-galactosidase from *Kluyveromyces lactis*) of 28,600 U/100 g lactose; temp., 37 ± 1 °C; and pH 6.9–7.0

Finally, the influence of selected salts dissolved in the reaction mixture was tested (Figs. 7 and 8). This factor affects the course of transgalactosylation reaction in two ways. Besides reducing the activity of water, certain substances may alter the enzyme activity. Research conducted by Pal et al. [45] indicated that cations Mn^2+^ and Mg^2+^ increase the activity of β-galactosidase from *K. lactis*. Monovalent cations such as Na^+^ and K^+^ exhibit slightly weaker promoting effect. Another study demonstrated that K+ has negligible impact on β-galactosidase activity. Furthermore, the addition of Mg^2+^ increases the enzyme activity, but only in an optimal concentration of ions [43]. Due to rising concentration of MgCl_2_ in the reaction mixture, the content of ascorbic acid galactoside increased even by 36% for 0.75 M MgCl_2_ (from 2.53 to 3.43% d.m.). However, a dramatic drop in the enzyme activity was noted for solution of 1 M MgCl_2_. Similarly to Mg^2+^ ions, K^+^ contributed to significantly improved yield (3.07% d.m. for 1 M KCl). The presence of 0.1 or 1 M NaCl diminished the final product content by a few percent (approx. 2.37% d.m.) while 0.1 M MnCl_2_ solution caused a decrease of about one quarter (1.93% d.m.). In the case of galactosyl derivative of gluconic acid, an increase by 17.5% was achieved after introducing NaCl compared with the reaction mixture without any salts [36]. The application of MgCl_2_ solution resulted in the fall of the final derivative content (even by 12.9% for 1 M MgCl_2_). Interestingly, no distinct change was observed for synthesis in KCl solution. The presence of selected salts does not only increase the yield of product but also might cause some difficulties in separating the derivative from the post-reaction mixture as preparative chromatography and ion exchange is used for the purification process. Setting conditions for the effective product isolation will be the aim of the next research cycle.

These findings emphasize the necessity of adjusting conditions of transgalactosylation reaction independently for different acceptors.

### Yield of the Ascorbic Acid Derivative Synthesis

The content of ascorbic acid galactoside amounted to 2.53% d.m. (12.7 g/L) after 9 h of reaction under optimal conditions without any additional salt. The elongation of time seemed to lead to further-reaching hydrolysis of galactoside—a sample taken after 24 h reaction contained only approx. 2.1% d.m. Maximum concentration was noted for solution of 0.75 M MgCl_2_ (3.43% d.m., 17.2 g/L, after 9 h). As a comparison, Donpou et al. [20] synthesized 6-*O*-β-galactopyranosyl-l-ascorbic acid with β-galactosidase from *A. oryzae* with a yield of approx. 36 g/L and Kawanaka et al. [21]—approx. 42 g/L (β-galactosidase from *B. circulans*). Hashimoto et al. [17] produced approx. 61 g/L (as above) of 6-*O*-α-d-galactopyranosyl-l-ascorbic acid utilizing α-galactosidase from *C. guilliermondii* H-404 and melibiose as a substrate (all values estimated on the basis of data from patents). Taking into account ascorbic acid glucosides, Hsieh et al. [4] obtained about 18 g/L of this derivative when mycelia of *A. niger* were used as a catalyst (and maltose as a donor). Zhang et al. [9] reported the yield of 13 g/L in case of 2-*O*-α-d-glucopyranosyl-l-ascorbic acid (AA-2G) synthesis as a result of α-cyclodextrin glucanotransferase (from recombinant *E. coli*) activity. Nevertheless, glucoamylase treatment was necessary to receive AA-2G from AA-2-oligosaccharides.

Obviously, the post-reaction mixture in the presented case consisted also of unreacted substrates and other products of transgalactosylation process as well as lactose hydrolysis. For instance, after reaction in a 50% (*w*/*v*) solution with sodium ascorbate and lactose at the molar ratio of 1.9:1 and the enzyme dose of 28,600 U/100 g lactose, the following substances (besides galactoside) were identified: disaccharides (mainly lactose), 7.8% d.m.; GOS, 2.7% d.m.; galactose, 14.8% d.m.; glucose, 28.3% d.m.; and unreacted sodium ascorbate as a remainder.

## Conclusions

After 9 h reaction conducted in 50% (*w*/*v*) mixture of sodium ascorbate and lactose in the molar ratio of 1.9:1, with enzyme dose of 28,600 U/100 g lactose and solution pH = 6.9–7.0, but without any added salt, the content of ascorbic acid galactoside reached approx. 2.5% d.m. Lower concentration of substrates caused the reduction in yield. Increasing or decreasing the amount of enzyme preparation or the ratio of donor and acceptor had an unfavourable impact on the course of reaction. The optimal pH range for transgalactosylation properties of β-galactosidase from *K. lactis* was consistent with that for hydrolytic activity. Introducing Mg^2+^ or K^+^ into the solution increased the product content, but the presence of Na^+^ or Mn^2+^ contributed to the opposite effect. Results achieved in this study may be found to be satisfying despite the low reaction yield compared with the available literature.
